# Identification by Teeth Casts

**Published:** 1905-02

**Authors:** 


### IDENTIFICATION BY TEETH CASTS.

A dentist has called attention to the value of teeth casts in identification—a means which does not seem to have previously been used. He relates the following example: A gentleman went to Africa to shoot big game. Soon a newspaper report appeared that he had been murdered by the natives. His property was large, but probate would not be granted on the basis of the newspaper report. His brother went to Africa and was shown the spot where the murdered man was said to be buried. The skeleton was found and brought to England. But how could it be identified? The probate court remained inexorable. It occurred to a relative that the man had consulted a dentist and the skull was brought to him. He had supplied the missing man with a set of artificial teeth and kept the cast of his mouth. Comparison of this with the skull at once proved his identity beyond doubt and probate was granted forthwith. The dentist insists that, as in the case of fingerprints, no two persons' mouths are alike and that a mouth may be identified by a cast taken years ago even though teeth have subsequently been destroyed or the gums lost as in a skeleton.—*Journal American Medical Association.*

